# Geomorpho90m, empirical evaluation and accuracy assessment of global high-resolution geomorphometric layers

**DOI:** 10.1038/s41597-020-0479-6

**Published:** 2020-05-28

**Authors:** Giuseppe Amatulli, Daniel McInerney, Tushar Sethi, Peter Strobl, Sami Domisch

**Affiliations:** 1 Yale University, School of Forestry & Environmental Studies, New Haven, CT 06511 USA; 2 Yale University, Centre for Research Computing, New Haven, CT 06511 USA; 3 Coillte Teoranta, Irish State Forestry Board, Plassey Road, Castletroy, Limerick, V94-C780 Ireland; 4 Spatial Ecology, 35A, Hazlemere Road, Penn, Buckinghamshire HP10 8AD United Kingdom; 5 European Commission, Joint Research Centre, Directorate for Sustainable Resources, Ispra, VA-21027 Italy; 6 Leibniz-Institute of Freshwater Ecology and Inland Fisheries, Department of Ecosystem Research, Müggelseedamm 310, 12587 Berlin, Germany

**Keywords:** Geomorphology, Hydrogeology

## Abstract

Topographical relief comprises the vertical and horizontal variations of the Earth’s terrain and drives processes in geomorphology, biogeography, climatology, hydrology and ecology. Its characterisation and assessment, through geomorphometry and feature extraction, is fundamental to numerous environmental modelling and simulation analyses. We, therefore, developed the Geomorpho90m global dataset comprising of different geomorphometric features derived from the MERIT-Digital Elevation Model (DEM) - the best global, high-resolution DEM available. The fully-standardised 26 geomorphometric variables consist of layers that describe the (i) rate of change across the elevation gradient, using first and second derivatives, (ii) ruggedness, and (iii) geomorphological forms. The Geomorpho90m variables are available at 3 (~90 m) and 7.5 arc-second (~250 m) resolutions under the WGS84 geodetic datum, and 100 m spatial resolution under the Equi7 projection. They are useful for modelling applications in fields such as geomorphology, geology, hydrology, ecology and biogeography.

## Abstract (summary)

**Table:** 

Measurement(s)	geomorphometric layer • geomorphometric feature • topography
Technology Type(s)	computational modeling technique
Factor Type(s)	elevation
Sample Characteristic - Environment	elevation
Sample Characteristic - Location	Europe • Asia • North America • South America • Africa • Oceania

Machine-accessible metadata file describing the reported data: 10.6084/m9.figshare.12145791

## Background & Summary

Geomorphometry is the science of quantitative analysis of the Earth’s surface^1^ and is amply used to address several multiscale geoscientific problems^2^. The primary inputs for such terrain analyses are remotely sensed Digital Elevation Models (DEMs), which provide an opportunity to derive a wide range of environmental variables and facilitates a better understanding of the patterns and processes in geomorphology, geology, climatology, hydrology or biodiversity science.

DEMs are usually categorised as being either a Digital Surface Model (DSM) or a Digital Terrain Model (DTM). The DSM is a 3D elevation model of the Earth’s surface that includes objects, such as trees and buildings, whereas the DTM represents the elevation of the bare earth without any temporary objects. DEMs can also be used to characterise topographical complexity^3^, as they provide the elevation above sea level, and allow for a wide array of geomorphometric metrics to be generated (also known as topographic, or geomorphometric variables). These parameters are quantitative measures of surface properties, which improve our understanding of the geographical, geomorphological and environmental properties of a given area of study^4^.

Topographical variations also influence numerous environmental dynamics, contributing significantly to the environmental complexity of a region. For example, they define the biotic and abiotic features at a sub-regional level^5^, and can shape the macro and micro climate of a given area^3^. The most common of these morphometric parameters, slope and aspect, can be used to further derive more complex features or curvature profiles of a terrain at any given location^6^. Florinsky^2,6^, in his monographs, makes accurate analytical and mathematical descriptions of 29 geomorphometric variables. Besides these, Sofia^7^ has written an extensive literature review of geomorphometry to advance the theoretical and practical understanding of geomorphological phenomena. Amongst all of the described geomorphometric variables, we selected 26 of the most commonly used ones.

Such topographic measures are also central to hydrological parameters shaping flow and erosion processes within the landscape, and to delineate catchment and stream features^8^. Additionally, the mapping and assessment of landform variability such as concavity and convexity is essential to obtain a better understanding of land erosion and landscape denudation dynamics in mountainous environments^9^. The morphometric properties of a surface are also important for predicting other phenomena such as wildfires^10^, mountain/alpine snow cover^11^ and landslide formation^12^. Understanding surface morphometric properties is also crucial beyond such geomorphological applications, since terrain features play a critical role in understanding contemporary biodiversity patterns given species occurrences (based on their habitat preferences) and potential species migration corridors where such detailed terrain information over large spatial extents is crucial.

Even with the latest DEMs, geomorphometric information can only be obtained on a case-by-case, location-specific basis. Therefore, standardisation is necessary to enable spatially comparative analyses between regions and continents. To this end, Amatulli *et al*.^5^ established a suite of 15 geomorphometric variables based on 7.5 arc-second (~250 m) Global Multi-resolution Terrain Elevation (GMTED)^13^ data. Building upon this work, we present in this paper the Geomorpho90m dataset^14–16^. Here, we extend the concept in Amatulli *et al*.^5^, by calculating a suite of 26 DEM-derived geomorphometric variables based on the Multi-Error-Removed Improved Terrain (MERIT) DEM at a spatial resolution of 3 arc-second (~90 m)^17,18^, which to-date is considered the best-effort in global DEMs^19,20^.

This newly-developed Geomorpho90m dataset^14–16^ of 26 variables hence provides the foundation for globally seamless, high-resolution studies. It consists of the following raster layers with standardised spatial extent and pixel resolution: slope, aspect, aspect sine, aspect cosine, eastness, northness, convergence, compound topographic index (also known as topographic wetness index), stream power index, first directional derivatives, profile and tangential curvature, second directional derivatives, elevation standard deviation, terrain roughness index, roughness, vector ruggedness measure, topographic position index, multiscale deviation and roughness, and geomorphologic forms. Through the use of the latest geocomputational methods and underlying DEMs, Geomorpho90m offers a marked improvement over previous datasets of this nature.

The quality and appeal of Geomorpho90m lies in the rigorous scripting procedures and tiling system that allowed for multi-core processing in a super computer. Additionally, the computation was performed using the Equi7 projection^21^, which minimises the pixel-level distortions that otherwise often occur when unprojected data (i.e. latitude-longitude in the World Geodetic System (WGS)) are treated as if they were a square raster under a cartesian coordinate system. The scripting procedures allow both multi-core processing and Equi7 reprojection, and are thus complex tasks that require advanced geocomputation programming skills. Therefore, for the sake of end-user expediency, we have undertaken these steps at the outset to offer an all-inclusive data product. Finally, to assess the quality of our work, we have compared the newly created variables with those generated from DEMs derived from 3D Elevation Program (3DEP) and Light Detection and Ranging (LiDAR).

## Methods

The Methods section is divided into three subsections that includes: (i) Source Data, that describes the MERIT-DEM used to produce the global geomorphometric layers, as well as other ancillary DEMs used to compare the main product; (ii) Projection and Tiling system, which was chosen to minimise computational errors from surface distortions; (iii) Derived Geomorphometric Variables, which describes each geomorphometric layer and its principal use in environmental modelling. Additional information on these variables and related procedures (software and scripting routines) can be found in Amatulli *et al*.^5^.

### Source data

This section describes the DEM used to compute the global gemorphometric variables and two additional DEMs used for comparison. The principal dataset employed in this study was the Multi-Error-Removed Improved Terrain (MERIT) DEM 3 arc-seconds (~90 m)^17^, which was used to extract geomorphometric terrain features. In addition, ancillary DEMs were used for the purposes of comparison, which included DEMs derived from Light Detection and Ranging (LiDAR) and data from the 3D Elevation Program (3DEP). The resulting geomorphometric variables consisted of a suite of 26 layers calculated from the source layers, which had been previously reprojected under the Equi7 projection.

#### Multi-Error-Removed Improved Terrain (MERIT) - DEM

Due to the lack of global high-resolution DEM obtained from a single data-sensor source, there have been several attempts to combine DEMs generated from multiple sensors^13^. One of the principal DEM sources is the Shuttle Radar Topography Mission (SRTM), which was acquired in February 2000 and provides a near-global coverage DEM of the Earth’s surface from 56°S to 60°N during an 11-day period. SRTM used a C-band radar system on board the space shuttle and relied on interferometry to generate the DEMs.

One of the characteristics of SRTM is that the short wavelengths associated with C-band radar is unable to sufficiently penetrate vegetated areas, in particular in forested areas^22,23^. Indeed, SRTM has been used as a DSM to estimate the height of vegetation canopies^24^. However, this effectively inhibits the creation of a correct DTM. The attenuation rate over forests is also affected by both the forest density and moisture content within the forest (e.g. more penetration is expected in drier forest types). In addition, as with any SAR instruments, the resulting dataset contains both speckle noise and gaps on steep slopes due to degraded accuracy caused by radar foreshortening and layover.

In recent years, many research initiatives have sought to improve the quality of DEM datasets derived from spaceborne products such as the SRTM and Advanced Spaceborne Thermal Emission and Reflection Radiometer (ASTER) DEM^25^. These improvements have largely focused on the removal of height errors and artefacts contained within them. One such dataset is the Multi-Error-Removed Improved Terrain (MERIT) - DEM 3 arc-seconds (~90 m)^17^, which sought to correct SRTM for absolute elevation biases using high-precision LiDAR elevation from ICESat and ancillary datasets as a reference.

The MERIT-DEM is based on the National Aeronautics and Space Administration (NASA) SRTM3 version 2.1, the Japan Aerospace Exploration Agency (JAXA) AW3D global high resolution 3D map (version 1) and the Viewfinder Panorama’s DEM. It removed inherent features or errors found in these products that included: stripe noise, absolute bias, tree height bias and speckle noise. Stripe noise was removed from the detection of unrealistic regular terrain undulations using a 2-D Fourier filtering technique. Absolute bias was corrected by calculating the difference between the DEM and the ICESat elevations^26^. Tree-height bias was estimated from the combination of tree density^27^ and tree height^28^ using ancillary layers, and by comparing the obtained MERIT-DTM to ICESat, which can deliver DSM as well as DTM estimates. The speckle noise was removed using an adaptive-scale smoothing filter^29^. Yamazaki *et al*.^17^ noted that after the error removal, areas mapped with ±2 m or better vertical accuracy increased by 19% and that previous DEMs contained slope distortions in many of the major floodplains.

The resulting MERIT-DEM product covers the land area between 90°N and 60°S at a spatial resolution of approximately ~90 m at the equator in the Plate Carrée projection on a WGS84 Ellipsoid, and is considered to be the optimal best-effort DEM that is currently available as free and open data on a global scale^19,20^. One of the unique aspects of our study is the reprojection of MERIT-DEM to Equi7 (based on a bilinear interpolation method and the projection parameters previously outlined), and the associated computation of geomorphometric variables with minimal distortions. This is a complex procedure, which has been completed and made available for the benefit of future research.

#### 3D elevation program - 3DEP

The U.S. Geological Survey’s National Map provides 3D Elevation Program (3DEP) products and services of standardised DEMs for the US, which were previously referred to as the National Elevation Dataset (NED)^30^. The 3DEP products are delivered using consistent datum, elevation units, coordinate reference system, and are distributed at different spatial resolutions (1/3, 1, and 2 arc-seconds), and mosaicked and edge-matched.

For the purposes of this analysis, the Seamless 1 arc-second (~30 m ground sampling distance) 3DEP DEM product was used (hereinafter 3DEP-1). It provides complete coverage of the US land area and partial coverage of Alaska. The source data products for 3DEP-1 include:

- LiDAR point cloud data collected in 2014, which meets 3DEP specifications for horizontal accuracy and pulse spacing, as well as the resulting bare earth DTMs.
- IfSAR Digital Surface Model (DSM), which is a 5-metre raster only available over Alaska.
- IfSAR ortho-rectified radar intensity image, which is radar reflectance imagery only available over Alaska.

Due to the nature of 3DEP, which is a LiDAR derived product for the conterminous United States, the elevation values can be considered the best available DTM at 1 arc-second. Prior to the computation of the geomorphometric variables, 3DEP-1 was reprojected to Equi7 using the projection parameters previously described. During the reprojection we set the pixel resolutions to 100 m to match and be comparable to the resolution of the Equi7 MERIT-DEM. However, in the manuscript we kept the nomenclature as 3DEP-1 and MERIT-DEM, which refer to the original DEM data sources.

#### Light detection and ranging (LiDAR)

Light Detection and Ranging (LiDAR) is an active remote sensing method that measures distances using a laser. The LiDAR sensor emits thousands of light pulses every second, and the scanner records the portion of the reflected light from the target. Given that the LiDAR sensors consist of a GPS and a precise timing unit, it is possible to calculate the exact 3-dimensional location of each point. LiDAR datasets are most commonly delivered in the LiDAR Aerial Survey format (LAS, or in its compressed format LAZ), and consist of 3-dimensional point clouds in a typical XYZ structure.

One of the main applications of LiDAR data is to generate gridded raster products that represent Digital Terrain Models (DTM) and DSM^31^. The digital model datasets are generated using algorithms that convert irregular point cloud datasets to gridded rasters^32^. In our study, we computed the DTM and DSM from LiDAR data, primarily to assess the quality of the tree-height correction applied to MERIT.

In order to evaluate the effect of tree height correction, we compared MERIT-DEM to DTMs and DSMs derived from two LiDAR datasets downloaded from the OpenTopography website^33,34^. Extensive forest cover was evident through a visual inspection in Google Earth, in conjunction with the forest cover map created by Hansen *et al*.^27^. The presence of forest canopy allowed for the creation of both DTM and DSM, which differed substantially in height. This in turn allowed for the comparison of the LiDAR-derived DTM with MERIT, as the latter excludes tree height.

### Projection and tiling system

The World Geodetic System (WGS) is a global reference system for geospatial information used in geography and in several satellite derived products. Often used for global representation of the Earth, it consists of a spherical coordinate system expressed in degrees and a standard ellipsoidal reference surface fixed to a datum. The datum for the WGS was established in 1984 and last revised in 2004, and is referred to as WGS84 or EPSG code 4326^35^.

Any kind of quasi-spherical surface represented in 2D (map format) over large extents inevitably suffers from three types of potential distortion: (i) length distortions; (ii) angular distortions; and (iii) areal distortions. All of these distortions are caused when the spherical coordinates of WGS84 are regarded as square grids (i.e. Plate Carrée or Mercator projections) and increase with a latitudinal gradient, with very high areal/length distortion values in the subarctic and Antarctic zones. Therefore, any environmental values affected by the distortion, degrade geographic analyses, especially in the northern hemisphere^36^. These distortions also result in local data oversampling when one projects generic satellite images to a regular raster grid^36^. The Grid Oversampling Factor (GOF) metric was devised to capture the local data oversampling effect, and indirectly quantify the overall distortion caused due to reprojection^36^. The GOF was then used as a baseline, against which multiple projections were compared to determine the effect of the distortions^36^.

**Fig. 1:**
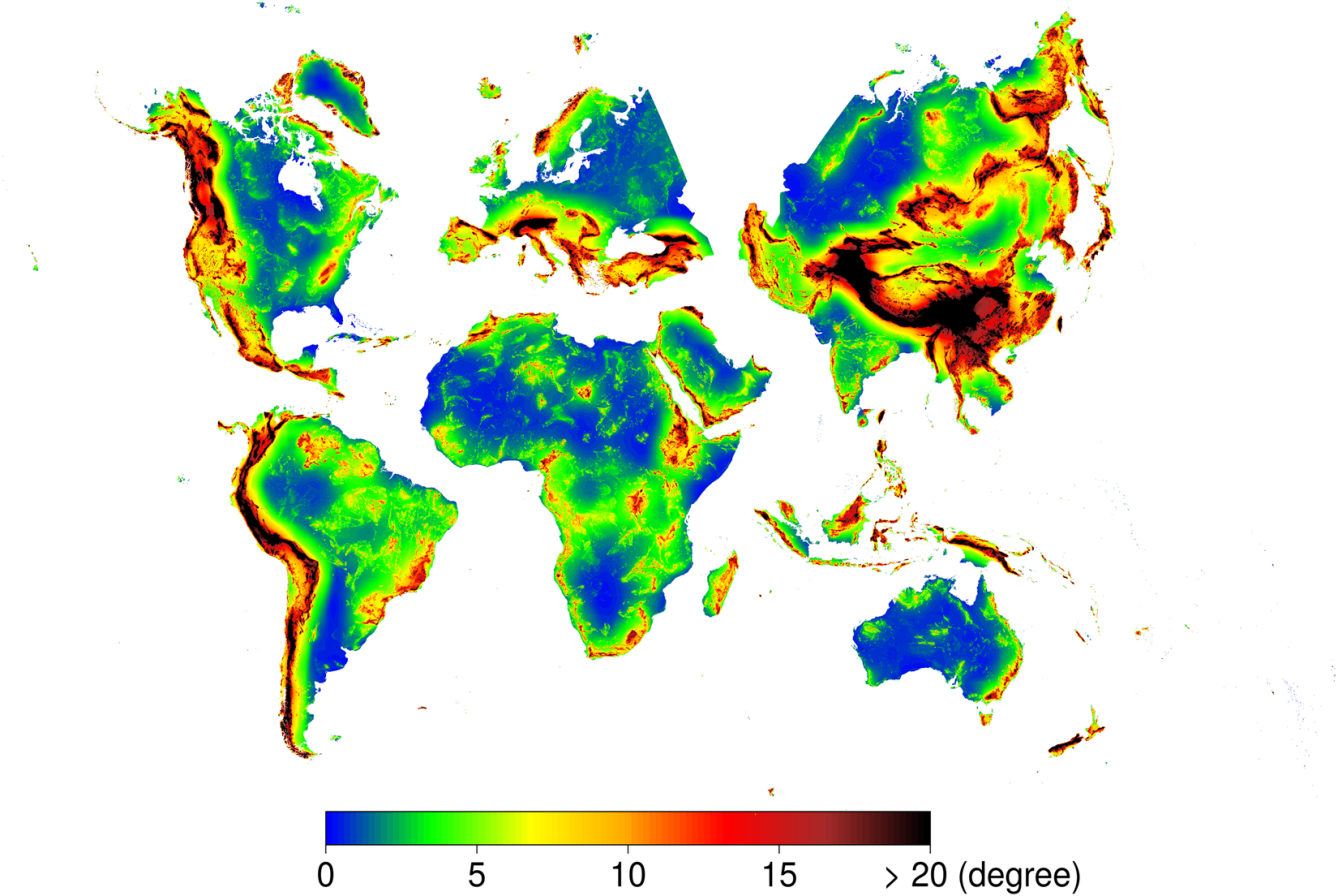
Maximum multiscale roughness. Quasi-global representation of the MERIT-derived maximum multiscale roughness (*rough-magnitude*), computed under Equi7 with a pixel size of 100 m using the computation parameters reported in the Multiscale roughness description. For the purpose of plotting the graphs, the 100 m resolution was aggregated to 1 km (10 × 10 pixels) by calculating the mean. Projection distortion of each Equi7 zone does not allow the merging of zones for a global continuous surface. However, the zones have been positioned adjacent to each other as a mosaic of six projections with gaps in between, to present a quasi-global map. Global visualisation in WGS84 for the other geomorphometric variables can be seen on the Geomorpho90m webpage^60^.

To minimise the overall areal and shape distortions in WGS84, the Equi7 Equidistant Azimuthal set of projections was proposed^36^ and is used in global mapping studies^37^. Bauer-Marschallinger *et al*.^36^ reported figures and tables for angular distortion and GOF. Generally, the Equi7 grids minimise the GOF to a global overall of ~1, compared to global and hemispherical grids which have a minimum GOF of 1.3. The Equi7 system was developed based on computation and end-user requirements, and consequently, the following objectives were carefully considered: firstly, landmasses should form compact, contiguous areas; secondly, the oceans should be used as borders between the sub-grids and finally, countries should not be split. As a result, the Equi7 Grid system covers the entire global with no gaps, and has a 50 km overlap between land borders. It is optimised for the storage and processing of global high resolution Earth Observation image data and consists of 7 projected continental sub-grids based on the Equidistant Azimuthal projection, which includes: Europe, Asia, North America, South America, Africa, Oceania and Antarctica (see Fig. 1 for a graphical representation of six sub-grids - excluding Antarctica). Additional information such as projection centres and GOF for each continental sub-grid can be found in Table 3 of Bauer-Marschallinger *et al*.^36^. Each sub-grid has the projection centre close to the continent’s barycentre and is divided into three nested tiling systems, which are T6 (600 km); T3 (300 km) and T1 (100 km)^36^. The predefined tiling systems are useful for the implementation of a multi-process workflow that does not require the information of the full continental zone. All Equi7 projection parameters, such as the seven continental central points, zone-continent borders, T6 tiles, etc., are stored in the Equi7 GitHub repository^38^.

A potential further reduction of projection distortions (linear, areal or angular), could be achieved by reprojecting the data into each of the 60 UTM grid zones and calculating the geomorphometric variables. Nonetheless, distortion increases in each UTM zone as the boundaries between the UTM zones are approached. Therefore, the enlargement of UTM tiles and consequently the weighted average as a function of UTM tile centre distance is required in order to avoid border effects. To achieve this solution requires complex programming and computation, especially considering the twice-computed pixels – once for each overlapped UTM tile, and the subsequently calculated weighted average. Such a complex exercise is only feasible when employing a high-resolution, e.g. a 30 m error corrected DTM, where it is important to preserve optimal accuracy.

From the perspective of algorithm development, the optimal approach is to calculate the geomorphometric variables using latitude and longitude in a spherical domain rather than treating the raster as a square grid. Unfortunately, all geomorphometric algorithms implemented in Geographic Information Systems (GIS) and remote sensing software packages treat the latitudinal-longitudinal rasters as square grids. Therefore, the only current solution is to reproject the raster to a grid system that minimises distortions.

We decided to use the Equi7 projection having considered its positive attributes as well as minor drawbacks. This decision was primarily driven by Equi7’s efficient data storage features, minimal area oversampling/distortion, and continental continuity^36^. In addition, we selected this scheme based on an assessment of the effect of slope values under WGS84 and Equi7. Our rationale for selecting the Equi7 is further discussed in the section “Evaluation of the geographic projection”.

### Derived geomorphometric variables

Geomorphometric layers, also known as topographic variables or topographic indices, can be derived from DEMs. The MERIT-DEM (as well as 3DEP-1 and LIDAR DEM) was used to calculate the 26 derived geomorphometric layers listed in Table 1. The derived layers were calculated based on a set of grid cells in the immediate vicinity of each focal cell, as defined by a moving window analysis. The size of the moving window was set to a 3 × 3 cell grid for most of the cases. Nonetheless, two layers (Multiscale deviation and Multiscale roughness) were calculated based on moving window size variations (more information is available in the variable description sections). Overall, the 26 variables can be grouped as those that: (i) quantify the rate of change (first derivative - 11 layers; second derivative - 5 layers), (ii) describe the ruggedness (9 layers), and (iii) identify the geomorphological forms (1 layer). We describe each single geomorphometric variable below, which also points to the acronym labelled in italics in the text below and reported in column 2 of Table 1. The variable acronyms also correspond to the file names stored in the data repositories^14–16^.

**Table 1:** MERIT-DEM derived geomorphometric variables.

Geomorphometric variable group	Geomorphometric variable name	Geomorphometric variable abbreviation	Software used
First order derivative	Slope	*slope*	GDAL: gdaldem^66^
	Aspect	*aspect*	GDAL: gdaldem^66^
	Aspect cosine	*aspect-cosine*	GDAL: gdaldem gdal_calc.py^66^
	Aspect sine	*aspect-sine*	GDAL: gdaldem gdal_calc.py^66^
	Eastness	*eastness*	GDAL: gdaldem gdal_calc.py^66^
	Northness	*northness*	GDAL: gdaldem gdal_calc.py^66^
	Convergence	*convergence*	GRASS GIS: r.convergence^67^
	Compound topographic index	*cti*	GRASS GIS: r.watershed^67^
	Stream power index	*spi*	GRASS GIS: r.watershed^67^
	East-West first order partial derivative	*dx*	GRASS GIS: r.slope.aspect^67^
	North-South first order partial derivative	*dy*	GRASS GIS: r.slope.aspect^67^
Second order derivative	Profile curvature	*pcurv*	GRASS GIS: r.slope.aspect^67^
	Tangential curvature	*tcurv*	GRASS GIS: r.slope.aspect
	East-West second order partial derivative	*dxx*	GRASS GIS: r.slope.aspect^67^
	North-South second order partial derivative	*dyy*	GRASS GIS: r.slope.aspect^67^
	Second order partial derivative	*dxy*	GRASS GIS: r.slope.aspect^67^
Ruggedeness	Elevation standard deviation	*elev-stdev*	PKTOOLS: pkfilter^69^
	Terrain ruggedness index	*tri*	GDAL: gdaldem^66^
	Roughness	*roughness*	GDAL: gdaldem^66^
	Vector ruggedness measure	*vrm*	GRASS GIS: r.vector.ruggedness.py^67^
	Topographic position index	*tpi*	GDAL: gdaldem^66^
	Maximum multiscale deviation	*dev-magnitude*	Whitebox: MaxElevationDeviation^68^
	Scale of the maximum multiscale deviation	*dev-scale*	Whitebox: MaxElevationDeviation^68^
	Maximum multiscale roughness	rough-magnitude	Whitebox: MultiscaleRoughness^68^
	Scale of the maximum multiscale roughness	*rough-scale*	Whitebox: MultiscaleRoughness^68^
geomorphological forms	Geomorphon	*geom*	GRASS: r.geomorphon^67^

The 26 geomorphometric variables (second column) are grouped based on their main mathematical operations (first column). The abbreviations in the third column correspond to the variable name of the file layer available for download.

#### Terrain first derivatives

In calculus, the first derivative is defined as the rate of change of a function, and in a geometric sense it is the slope of the tangent line of a function. When the first derivative is applied to a DEM, it represents the terrain slope of a relief. The terrain slope is the measure of steepness and is one of the most fundamental geomorphometric features that plays an important role in several natural phenomena such as soil accumulation, water infiltration, and snow depth, to mention a few. Slope can be computed in several ways in conjunction with vector direction. Details of each calculation are included below.

#### Slope

Terrain slope (*slope*), as mentioned above, is the rate of change of elevation in the direction of the water flow line. It is considered one of the most important terrain parameters and is often calculated first. It can be expressed in degrees or percentages, where for example, 5% means 5m of vertical displacement over 100m. It is especially important for the quantification of soil erosion, water flow velocity, or agricultural suitability^29^.

#### Aspect

Aspect (*aspect*) is the angular direction that a slope faces. It is expressed in degrees and therefore defined as a circular variable. We calculated the sine (*aspect-sine*) and cosine (*aspect-cosine*) of the aspect, changing a circular variable to a continuous variable, and allowing future processing, including reprojection under a bilinear algorithm or use them as continuous variables in regression analysis. The sine and cosine of the aspect, ranging from −1 to 1, can be used to emphasise differences in the north-south and east-west exposure^5^.

#### Eastness and northness

Using aspect and slope, we calculated northness (*northness*) and eastness (*eastness*). The sine of the slope when multiplied by the cosine of the aspect yields the northness, and when multiplied by the sine of the aspect provides the eastness^5,39^. Eastness and northness provide continuous measures describing the orientation in combination with the slope. In the northern hemisphere, a northness value close to 1 corresponds to a northern exposure on a vertical slope (i.e. a slope exposed to very low amount of solar radiation), while a value close to −1 corresponds to a very steep southern slope exposed to a high amount of solar radiation. Eastness and northness has been often used in plant species distribution and forest mapping^40^ and also in the spatio-temporal estimation of snow depth^41^.

#### Convergence

The convergence index (*convergence*)^42^ is a terrain variable that highlights the convergent areas as channels and divergent areas as ridges. It ranges from −100 for ridges to +100 for sink areas and 0 for planar or flat areas. In combination with the curvature parameters it is useful for delineating different landforms. The convergence index has been used in several studies regarding tree species distribution analyses and for down-scaling climate data over complex terrains^43^.

#### Compound topographic index

The compound topographic index (*cti*)^21^, also known as topographic wetness index, is computed as the logarithm of the cumulative upstream catchment area divided by the tangent of the local slope angle. This index is a proxy of the long-term soil moisture availability^44^. It is has been often used in applications that include species distribution modelling, species richness and composition, landslide susceptibility and soil carbon assessment^44,45^.

#### Stream power index

The stream power index (*spi*)^8^ is computed as the product between the upstream catchment area and the tangent of the local slope angle. The stream power index reflects the erosive power associated with flow and the tendency of gravitational forces to move water downstream^8^. It is commonly used in soil erosion models, landslide susceptibility and groundwater estimation.

#### First directional derivatives

Directional derivative (*d*) is the rate of change of the elevation in a specific direction. In particular the East-West first order partial derivative (*dx*) is the slope in an East-West direction, while the North-South first order partial derivative (*dy*) is the slope in a North-South direction. The first directional derivatives (dx and dy) can be used to estimate overland water flow and sediment flow by means of the SIMWE model^46^. Moreover, directional slope has been used to detect artefacts (voids, pits, sinks, sensor stripes) in DEMs^19^, due to its sensitivity to systematic noise such as striping, or artefacts such as voids and pits, in the DEMs.

#### Terrain second derivatives

In calculus, the second derivative is the derivative of a derivative. In other words, it is the rate of change of the slope and represents the curvature or concavity of a function. When the second derivative is applied to a DEM it represents the rate change of slope or aspect in a particular direction. The unit of curvature is radians per metre, where positive and negative values indicate convex and concave surfaces, respectively. Terrain curvatures directly affect soil erosion and composition, water accumulation and infiltration, and therefore indirectly drive the presence and composition of flora and fauna. Terrain curvatures can also be used as input parameters for hydrological and soil erosion modelling.

#### Profile and tangential curvature

Profile curvature (*pcurv*) measures the rate of change of a slope along a flow line, and affects the acceleration of water flow along a surface^29^. The tangential curvature (*pcurv*) measures the rate of change perpendicular to the slope gradient and is related to the convergence and divergence of flow across a surface^29^. The analysis of curvatures allows one to understand how water and sediments move through the landscape and helps to quantify their accumulation or dispersal^7,47^.

#### Second directional derivatives

The second directional derivative is the rate of change of the slope in a predetermined direction: the East-West second order partial derivative (*dxx*) is the derivative of a slope in a East-West direction, while a North-South second order partial derivative (*dyy*) is the derivative of the slope in a North-South direction.

#### Terrain ruggedness

Surface roughness is a common topographic attribute and is frequently measured using DEMs. It describes the ruggedness and topographic complexity (elevation variability) of landscapes within an area. Roughness maps are derived by measuring topographic variability around each grid cell in a moving window approach. The roughness is scale-dependent as a function of the moving window size and will significantly influence the final roughness map^48^. Described below are five indices computed using a classic 3 × 3 moving window approach, and two roughness indices obtained from a multiscale analysis, by progressively increasing the moving window size.

#### Elevation standard deviation

Standard deviation (*elev-stdev*) is a measure of the amount of variation within a dataset. The standard deviation of elevation was calculated using a 3 × 3 moving window. Values close to 0 indicate no variation, (i.e. flat areas), while areas with high standard deviation indicate areas with very steep terrain.

#### Terrain ruggedness index

The terrain ruggedness index (*tri*) is a mean of the absolute differences in elevation between a focal cell and its 8 surrounding cells. It is a type of statistical variance of elevation change across the 3 × 3 cells^49^. Flat areas have a value close to zero, while mountainous areas have positive values that can be greater than 500 m.

#### Roughness

Roughness (*roughness*)^50^ is expressed as the largest inter-cell absolute difference of a focal cell and its 8 surrounding cells. It is expressed in unit length, in our case metres, and is always positive, ranging from zero values in flat areas to progressively larger positive values in mountain areas. This variable is a measure based on a maxima, therefore it is more sensitive to the artefacts that remain in the MERIT-DEM.

#### Vector ruggedness measure

The vector ruggedness measure (*vrm*)^51^ quantifies terrain ruggedness by measuring the variation by means of sine and cosine of the slope in the three-dimensional orientation of grid cells, within a moving window. Slope and aspect are decomposed into 3-dimensional vector components (in the x, y, and z directions) using standard vector analysis in a user-specified moving window size (3 × 3). In other words, it captures variability of slope and aspect in a single measure. The vector ruggedness measure quantifies local variation of slope in the terrain more independently than the topographic position index and terrain ruggedness index methods^51^. It is dimensionless because of sine-cosine derivation, and values range from 0 to 1 in flat to rugged regions, respectively.

#### Topographic position index

The topographic position index (*tpi*)^29,52^ is the difference between the elevation of a focal cell and the mean of its 8 surrounding cells. It ranges from positive to negative values and they correspond to ridges and valleys, respectively. Zero values correspond to flat areas.

#### Multiscale deviation

The deviation from mean elevation (*dev*) is a unitless measure of topographic position, and the difference between the elevation of the centre cell and mean elevation divided by the standard deviation of the entire window^53^. The deviation from the mean elevation range is unbounded (−∞, +∞), and a positive or negative sign indicates whether the central cell is above or below the surrounding mean elevation. Furthermore, the magnitude value indicates the relative spread of the elevation distribution in its surrounding area^54^. The multiscale analysis of the deviation consists of the estimation of spatial patterns using a range of window sizes. The maximum value of the multiscale deviation identifies the Maximum Elevation Deviation value (*dev-magnitude*) and the window size (*dev-scale*) where the maximum value is depicted^53^. To calculate the multiscale deviation over a range of spatial scales we vary the moving window dimensions ranging from 3 × 3 to 4001 × 4001 grid cells, by a constant increment of 3 grid cells. To our knowledge this is the first time that the multiscale deviation variables have been calculated at global scale.

#### Multiscale roughness

The multiscale roughness^55^ (*rough*) is the spherical standard deviation (*σ*_*s*_) of the sum of 3-dimensional vector components derived to calculate the *vrm*. Its units are degrees. It can be computed in moving windows of different sizes, and in case of a 3 × 3 mowing window, it corresponds to *rough* = *σ*_*s*_(*vrm*). Lindsay *et al*.^55^ described the analytical aspect and computation parameters in detail. If these deviations are large, the surface is rough. On the other hand, if they are small, the surface is smooth^48^. The multiscale roughness variation follows a similar distribution to the multiscale deviation and its maximum values (*rough-magnitude*) identify magnitude and scale (*rough-scale*). To calculate multiscale roughness over a range of spatial scales, we vary the moving window dimensions ranging from 3 × 3 to 4001 × 4001 grid cells, by a constant increment of 3 grid cells (internal iterations). Likewise, to our knowledge this is the first time that the multiscale roughness variables have been calculated at global scale at 90 m resolution. A quasi-global representation of the MERIT-derived maximum multiscale roughness, is depicted in Fig. 1. From a computational perspective, the multiscale roughness and deviation variables have been the most difficult to calculate. This was due to the wide tile overlapping (>4001 grid cells), useful to avoid border effects, and due to internal iterations that retain the maximum values. We decided to plot the *rough-magnitude* in Fig. 1 as a quasi-global visualisation, due to its novelty and practical utility as locally adaptable and scale-optimised analyses for mapping applications^55^.

#### Geomorphological forms

##### Geomorphon

The geomorphological forms (*geom*) consist of 10 classes that can be extracted from DEMs using morphometry techniques^56^. This technique identifies geomorphological phonotypes also known as geomorphons. It is based on pattern recognition rather than differential geometry and thus has high computational efficiency. It classifies the terrain in terms of the following features: flat, peak or summit, ridge, shoulder, spur, slope, hollow, footslope, valley, and pit or depression (class label-number for each geomorphon are respectively: 1,2,3,4,5,6,7,8,9,10; schematic representation in Figs. 3 and 4b of Amatulli *et al*.^5^). These geomorphon classes have been used in a wide range of studies such as landslide susceptibility mapping^57^, human mobility^58^, and ecosystem service assessments^59^. Other variables can be derived by the analysis of the geomorphon shapes: intensity, range, variance, extend, azimuth, elongation, width^56^. As they are considered experimental, we decided do not compute these variables.

A static global visualisation of the geomorphometric variables under WGS84 geodetic datum can be seen on the Geomorpho90m webpage^60^. Additionally, more detailed patterns are shown in Fig. 5 of Amatulli *et al*.^5^. Moreover, the correlation amongst all geomorphometric variables is shown in the correlation matrix reported in Figure 7 of Amatulli *et al*.^5^. Even if the underlying DEM has a coarse resolution of 250 m, the correlation and general pattern will be similar to the 90 m resolution variables. The matrix can be used to select variables in accordance with the concerned case study and as a function of the employed modelling technique.

## Data Records

### Data repository

The Geomorpho90m dataset is a set of gridded layers stored as GeoTIFF files. It is derived from the 90 m MERIT-DEM, and is global in extent (60°–85°N latitude), including all continents except for Antarctica. Subsets of the 26 geomorphometric variables can be visualised on OpenLandMap WebGIS^61^ under the thematic layers “Relief/Geology”. These layers can also be downloaded as three different products:

- at 100 m resolution, under Equi7, available to download at Spatial-Ecology repository^14^.
- at 3 arc-seconds (~90 m) resolution, under WGS84, available to download at OpenTopography repository^15^.
- at 7.5 arc-seconds (~250 m) resolution, under WGS84, available to download at PANGAEA repository^16^.

### File nomenclature

The file name identifies: the geomorphometric variable abbreviation (see Table 1), the spatial resolution, the DEM source (MERIT) and the tiling system, within the following structure:

*variable abbreviation_resolution_DEM source layers_tiling system.format*

The layers under the Equi7 projection are considered to contain the most reliable values owing to minimal geographic distortion. For our Geomorpho90m dataset we use the T6 tiling system and tile nomenclature (more info at the Equi7 github^38^) proposed by Bauer-Marschallinger *et al*.^36^. We encourage users to use the Equi7 projection especially for studies at a continental or global scale.

Below are two examples of the layer names under Equi7:

- *tri_100M_MERIT_AF_006_066.tif*: layer showing the terrain ruggedness index at a 100 m spatial resolution in Equi7 stemming from the MERIT-DEM in Africa with the tile position 006_066.
- *rough-magnitude_100M_MERIT_AF_006_066.tif*: layer showing the maximum multiscale roughness at the identical location (tile position 006_066).

The majority of users utilise the WGS84 geodetic datum, and hence we reprojected the layers from Equi7 to WGS84 at a 3 arc-second (~90 m) spatial grain. Here, we used the tiling system implemented in the MERIT-DEM dataset (more info at MERIT website^18^). Each tile covers a 5 × 5 degree (6000 × 6000 cell) extent, while the tile name describes the position of the lower left pixel of the layer.

Below are two examples of the layer names under WGS84:

- *slope_90M_MERIT_s30e125.tif*: layer showing the slope at a 3 arc-second spatial resolution in the WGS84 stemming from the MERIT-DEM in Australia with the tile position s30e125.
- *aspect_90M_MERIT_s30e125.tif*: layer showing the aspect at the identical location of the *slope_90M_MERIT_s30e125.tif*.

In addition, each geomorphometric layer at a spatial resolution of 3 arc-seconds and 100 m was stored as 32-bit floating point (Float32 data type) for maximum precision, allowing for the computation of other customised variables (e.g., coefficient of variation) or for aggregating the layers at coarser spatial resolutions for macro-scale environmental models.

For the OpenLandMap WebGIS^61^ visualisation, we reprojected the layers from Equi7 to WGS84 (EPSG:4326 code^35^) with a spatial resolution of 7.5 arc-second (~250 m), stored as 16-bit integer (Int16 or UInt16 data type; scale factor is reported in the GeoTIFF metadata) to enable a fast visualisation rendering.

## Dataset comparison

The quality of the underlying DEMs, in terms of the vertical and spatial accuracy, directly influences the quality of the newly-developed geomorphometric layers. In this section we assess the sensitivity of the geomorphometric layers with respect to DEM accuracy and we describe it in three subsections organised as follows: i) Evaluation of the geographic projection, where we show the possible artefacts that could stem from computing the geomorphometric variable under WGS84 geodetic datum and the Equi7 projection; ii) MERIT-DEM versus 3DEP-1-derived geomorphometric layers, where we describe the divergence of the most common geomorphometric layers obtained from MERIT-DEM and 3DEP-1 DEMs; iii) MERIT-DEM vs LiDAR elevation comparison, which outlines the influence of removing the tree height bias in MERIT-DEM using the DTM and DSM obtained from LiDAR. Overall, these analyses highlight the quality of the MERIT-derived geomorphometric layers and make it possible to identify potential errors in DEMs.

### Evaluation of the geographic projection

Cartographic projections are required to map the Earth’s surface on a 2-D plane and this is particularly relevant for DEMs. Regardless of the projection used, some type of distortion will occur in the resulting map but the selection of a suitable projection should, in principle, minimise the extent and type of distortions^36^. In general, map distortions diminish as the geographic area is reduced, for instance, when moving from global to continental or regional scales. Moreover, distortions increase as one travels along a surface, away from the projection centre. This distortion is an unavoidable property of map projections, and it is important to assess its effect on any type of spatial analysis, particularly on those carried out on a large scale. To assess the effect of map distortions on the geomorphometric variables, we analysed the slope variations under two geographic locations having different surface distortions.

The slope is defined as the rate of elevation change along the direction of the water flow, and calculated using a 3 × 3 cell moving window. The rate of change can be expressed as a percentage of elevation change over 100 metres. In order to have the same weight in the *x* and *y* directions on the rate of change, the cell size must have the same dimension in the *x* and *y* directions. This is not the case when the Geographic Coordinate System is used and specifically where the longitudinal gradient (in *y* dimension) stays constant with respect to the latitudinal gradient (in *x* dimension), which decreases away from the equator.

Since terrain slope is one of the most widely used geomorphometric variables, all GIS and remote sensing software have adopted algorithms to compute it. However, none of these algorithms employ a correction procedure to account for grid distortion in the *x* and *y* dimension. In other words they treat latitude, longitude data as a matrix on a square grid. Instead, it is established that meridians converge towards the Poles and parallel circles’ distance varies only slightly. Thus, a square grid at the Equator becomes a rectangular grid at higher latitudes. Therefore, to quantify the influence of changes in latitudinal dimension of *x*, we compared slope values under WGS84 with those under Equi7 for two study areas of 500 × 500 grid cells, with MERIT-DEM as a static base layer. This procedure can be shown using a simulated DEM under two distinct locations, one in the subtropical zone and the other in the subarctic zone. Nonetheless, to demonstrate a real potential effect, we select only one zone from MERIT-DEM.

**Fig. 2:**
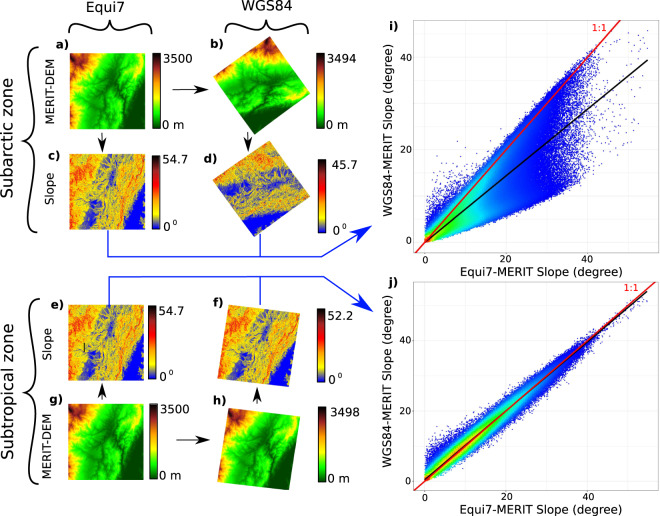
Projection bias assessment. Graphical representation of the difference in terrain slope calculation due to the effect of using the World Geodetic System 1984 (WGS84) (raster panels right-hand side) compared to the Equi7 projection (raster panels left-hand side). A study area located in the subtropical zone (image centre: longitude −83.26, latitudes 9.05) was used to subset the MERIT-DEM for an area of 500 × 500 grid cells (*g*). This area has been transposed to a subarctic zone (image centre: longitude −38.19, latitudes 72.80) under the Equi7 projection (**a**). After having been reprojected to WGS84 (*b,h*), the variable slope was calculated in the four conditions (**c–f**), and then reprojected back to Equi7 for comparisons (see blue line-arrows). The scatter plots on the right-hand side shows the WGS84-MERIT slope (**d,f**) vs. the MERIT slope under the Equi7 projection (**c,e)**, respectively for the subarctic zone (**i**) and for the subtropical zone (**j**). The red lines represent the 1:1 relationship and black lines represent a linear model between the two axes. The slope calculated under WGS84 in the subarctic zone is clearly underestimated compared to the one calculated under the Equi7 projection.

The graphs presented in Fig. 2 show the difference in slope calculations as a direct result of using latitude and longitude on WGS84 with those from a square grid on Equi7. To compare the same MERIT-DEM under WGS84 and Equi7 at two distinct locations we transpose (i.e., Equi7 shift coordinates - note: no-reprojection) the subtropical zone MERIT-DEM (image centre: longitude -83.26, latitudes 9.05; in Costa Rica) (Fig. 2g) to a subarctic zone (image centre: longitude -38.19, latitudes 72.80; in Greenland) (Fig. 2a), under Equi7. This produces a simple displacement along the north-south axis without changes in the elevation pixel value (no-interpolation). After reprojecting the MERIT-DEM to WGS84 (Fig. 2b,h), we calculated slope (Fig. 2f,d) and subsequently reprojected it back to Equi7 (blue line-arrows in Fig. 2) to compare the results using the scatter plots. Figure 2i corresponds to the slope correlation of the subarctic zone, whereas Fig. 2j corresponds to the subtropical zone). In each scatter-plot (Figs. 2i,j), the red line represents a 1:1 relationship, while the black line is a fitted regression model between the variables. The variations in slope calculations between the two systems are minimal within the subtropical zone (Fig. 2j), as the study area is adjacent to the equator. However, in the subarctic zone, the variations are significantly different, as the slope calculated under WGS84 is underestimated compared to Equi7 slope. This is because the east or west-facing slopes will have their gradient significantly underestimated due to the stretching of *x* dimensions in the east-west direction (note all the points notably beneath the red line of Fig. 2i). On the other hand, the north- and south-facing slopes may be moderate correctly estimated (note all the points close to the red line of Fig. 2i).

Similar to slope, all geomorphometric variables are influenced by underlying grid distortions. In particular, the slope is influenced by both length and angular distortions, as are all of the other geomorphometric variables listed under the first and second derivatives group. In contrast, the ruggedness geomorphometric variables are influenced more by areal distortions because of elevation differences at the pixel level. These results emphasise the importance of computing the geomorphometric variables under the Equi7 projection.

In conclusion, it is not that the WGS84 geodetic datum is wrong and distorted but its treatment of latitudinal and longitudinal grids as squares is erroneous, as in the Plate Carrée projection. Consequently, the calculation of any geomorphometric variables under WGS84 should be avoided.

### 3DEP-1 versus MERIT-DEM comparison

For geomorphometrical and hydrographical applications, the elevation difference between two DEMs is important since any application will be contingent on the values of the derived geomorphometric variables, for example, impacting on the delineation of streams and catchments. In the following sections, we analyse the difference in the elevation values between the 3DEP-1 and MERIT DEMs, as well as their derived geomorphometric variables. The 3DEP-1 is a LiDAR-based DEM and given its high accuracy, can be used as a reference elevation that has negligible errors. Initially, we compare the elevation difference between 3DEP-1 and the MERIT-DEM, and subsequently we analyse how the differences in DEMs influence the derived geomorphometric variables.

#### Comparing DEMs using the Elevation Deviation Index (EDI)

The elevation difference, or deviation, at pixel level between two DEMs can be expressed as

1 $${\overrightarrow{\Delta }}_{i}={x}_{i}-{y}_{i}$$

where **x** and **y** are the elevation values in each single pixel *i*. The ${\overrightarrow{\Delta }}_{i}$ value is equal to 0 if the two DEMs have the same elevation. The overall raster of ${\overrightarrow{\Delta }}_{i}$ values represents the Δ surface.

To identify areas where the deviation is stronger, the deviation at each pixel needs to be considered with the surrounding elevation pixel values (**x**_*i*+1_). In our case, we label 3DEP-1 as *y* and MERIT-DEM as *x*. Therefore, considering a circular window of 23 × 23 pixels that slides across *y*, it is possible to obtain the standard deviation, which estimates the local elevation roughness. Mathematically, the standard deviation of *y*_*i*_ in a moving circular window is expressed as:

2 $${\sigma }_{i}=\sqrt{\frac{1}{N-1}\mathop{\sum }\limits_{i}^{N}\,{({y}_{i}-\hat{y})}^{2}}$$

If we integrate the ${\overrightarrow{\Delta }}_{i}$ and its surrounding standard deviation, we obtain the Elevation Deviation Index (*EDI*), which is defined as the ratio

3 $$EDI={\overrightarrow{\Delta }}_{i}/({\sigma }_{i}+k)$$

*EDI* represents the relative deviation over the surrounding elevation variability in the moving window. The component *k*, in above equation, is used to prevent the situation that areas completely flat, with *σ*_*i*_ = 0, will produce infinite values of the *EDI*. In our case, we set *k* = 0.1, which is a very small value compared to the calculated *σ*_*i*_, even in quasi-flat areas. *k* does not influence the *σ*_*i*_ and consequently the overall performance of the *EDI*.

**Fig. 3:**
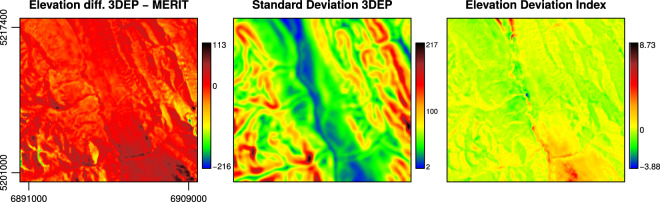
Elevation Deviation Index. Elevation Deviation Index *EDI* (*c*) obtained as the ratio of elevation difference (*a*: 3DEP-1 - MERIT) and elevation standard deviation calculated using a moving window of 5 × 5 pixels of the 3DEP-1 (*b*). The coordinates reported in *a* are in Equi7 and expressed in metres. The study area refers to a zone of 18.4 × 20 km, which has a high level of forest cover, and is located in Alberta, Canada, close to Jasper National Park - image centre 118.25°W 53.29°N. The same area is used in the Fig. 4 to assess the geomorphometric variables.

For example, a local elevation difference of 1 m will create a higher index in flat areas compared to mountain regions; and the index can be positive or negative with respect to the ${\overrightarrow{\Delta }}_{i}$. The *EDI* can be used to select zones where the elevation difference between the 3DEP-1 and the MERIT-DEM is substantial considering the roughness of the surrounding areas. Hence, flat areas will be more sensitive to the *EDI* compared to steep, mountainous areas. We expect that areas with extreme *EDI* will be more prone to deviating stream networks compared to zones with *EDI* close to 0 (see Fig. 3).

When comparing DEMs with unknown or significant errors, the standard deviation of the mean of the two DEMs can be calculated. Besides, this standard deviation is a measure of roughness, and the window size (Eq. 2) reflects neighbouring influences. A large window size will produce a larger standard deviation and thus lower *EDI*, on average. The moving window size can be adjusted with respect to the resolution of the DEMs or on the basis of the surrounding roughness. The *EDI* can be applied on a global scale by comparing different DEMs, and highlighting areas where the DEMs have discrepancies.

Figure 3 shows *EDI* and its components for an area of 18.4 × 20 km. The extreme Δ_*i*_ values (black and blue areas in *a*) do not necessarily produce extreme *EDI*. With respect to the *EDI*, in the largest part of the study area, the DEMs are in agreement (yellow - green colour) and located in zones with a high level of roughness. On the contrary, in flat areas (blue colour in b) the *EDI* can reach extreme values (blue and dark red colour in c).

#### Comparing the continuous geomorphometric variables

To compare the geomorphometric variables derived from the 3DEP-1 and MERIT-DEM under the same scale unit, we normalise the difference expressed as a Δ surface. Hence, we deal with the difference (for example pcurv-3DEP-1 - pcurv-MERIT) by scaling all positive values to fall between 0 and +1, and negative values to fall between -1 and 0. As a result, the difference value at 0 remains at 0 when scaled (e.g. 0, 9 scaled to 0, 1; −3, 0 scaled to −1, 0).

Consequently, the normalised Δ surface derivative from each geomorphometric variable can be compared having the same unit and can be used to assess the sensitivity of the variables to the differences in DEM elevation. In fact, in instances where the Δ surface has a value close to 0, this suggests that a geomorphometric variable is not strongly influenced by the difference between the two DEMs. In contrast, in instances where the Δ surface has several pixels with negative or positive values, this means that they are influenced by the DEM’s difference.

**Fig. 4:**
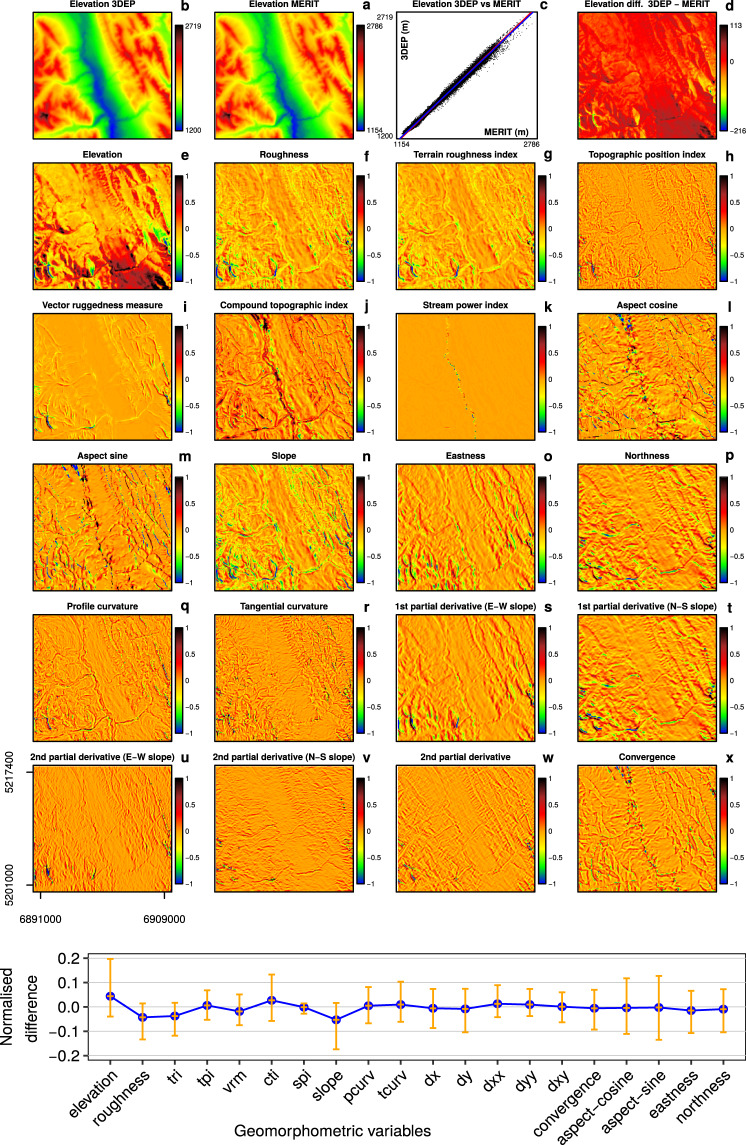
Normalised difference maps. Normalised difference maps represented as Δ surface, for each geomorphometric variable derived from 3DEP-1 minus MERIT-DEM. To compare the geomorphometric variables under the same scale unit, the difference has been scaled from -1 to 1 (minimum and maximum stretching) keeping the 0 value (no difference) at the 0 position. The bottom plot reports the normalised difference as mean (blue line) and standard deviation values (orange vertical lines) of the maps. The mean and standard deviation plot helps to identify which geomorphometric variables are more sensitive to variation in the DEMs (e.g. high sensitivity for variables derived from slope and aspect). The coordinates *u* in Equi7 are expressed in metres and refer to a study area of 18.4 × 20 km, which has a high level of forest cover, and is located in Alberta, Canada, close to Jasper National Park - image centre 118.25°W 53.29°N.

Figure 4 shows an overview of the normalised Δ surface for each geomorphometric variables. Two elevation plots (Fig. 4a,b) show the 3DEP-1 and MERIT DEMs and relative scatter plot (Fig. 4c). The elevation difference (Fig. 4d) shows values ranging from −113 m to +216 m. The largest values of difference are located close to the peak areas, and the smallest values are concentrated in the valley areas. Figure 4e shows the normalised version. In contrast, the other plots show the spatial variability of the geomorphometric difference, expressed with normalised values. Values close to −1 and +1 mean high sensitivity to elevation difference, and conversely, values close to 0 mean less sensitivity. In general, the overall correlation between 3DEP-1 and MERIT-DEM is very high, with the blue line representing a fitted regression model, which is very close to the 1:1 red line. These results are in line with other studies that evaluate the accuracy of the MERIT-DEM^17,19^.

The differences in elevation are mainly due to the radar beam’s shadow, which is usually evident in steep terrain. In fact, in Fig. 4d, the area with high elevation difference is located in the south-west corner with Δ values larger than 200 m. The greatest relative deviations are in high-relief areas for most of the geomorphometric variables except for the compound topographic index, convergence, and sine and cosine of aspect (see Fig. 4j,x,l,m). For these exceptions, the relative deviations are greatest in low-relief areas, especially in the valleys. Indeed, flat areas are very sensitive to the DEMs accuracy and slight variations in the elevation can switch the aspect to the opposite direction.

The behaviour between the compound topographic index (*cti*) and stream power index (*spi*) differs due to the logarithmic scale used in the *cti*. Consequently, the deviation of the *cti* is visible when there is a small variation of the flow accumulation. On the other hand, the *spi* does not employ a logarithmic scale, and the deviation is only evident when there is a drastic change in the flow accumulation areas that are adjacent to stream locations.

Visually, the aspect-sine, the aspect-cosine, slope, eastness, northness and the convergence are geomorphometric features that are very sensitive to differences between the two DEMs (see Fig. 4f,g,h,x).

To support the visual assessment of the maps in Fig. 4 with numerical values, we analysed the normalised Δ surface by plotting the mean values and standard deviation for the positive and negative values (see Fig. 4). In fact, similar patterns can also be seen for any of the aforementioned variables with high standard deviation (see vertical lines). The aspect is very sensitive to DEM differences in both steep terrain and flat areas. Even the derived sine and cosine Δ surface show black and blue areas (+1 and −1, respectively) in the central valley (see Fig. 4i,m). Note that these areas are not apparent in the other variables. Just as 1st partial derivatives have been used to detect artefacts in DEMs^19^, the Δ surface of the aspect-sine and aspect-cosine can be used to highlight areas where the two DEMs show differences in elevation.

#### Comparing the categorical geomorphometric variables

**Fig. 5:**
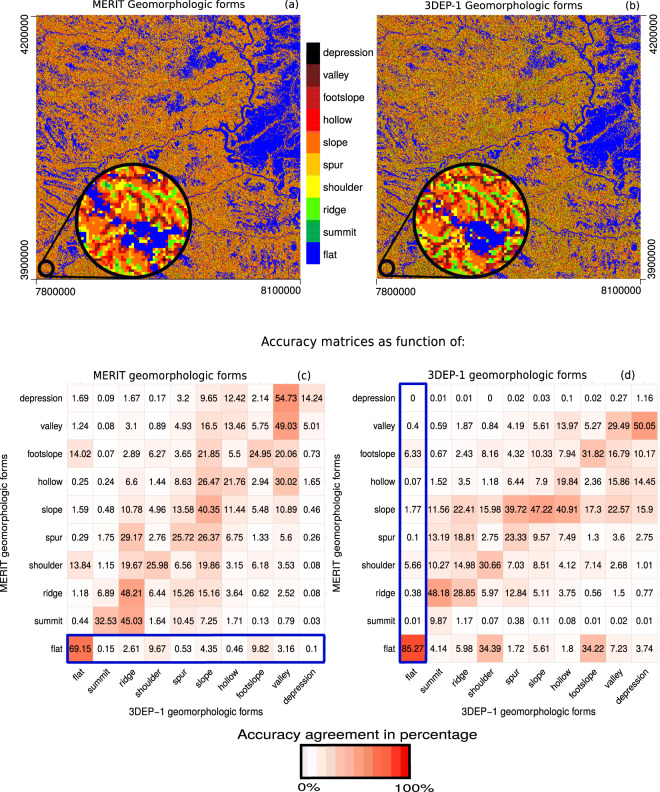
Geomorphological forms maps and confusion matrices. The geomorphological forms have been computed for a study area of 3000 × 3000, 100 m pixels in South Dakota (USA) derived from MERIT (**a–c**) and 3DEP-1 (**b–d**), respectively. The confusion matrix values are expressed in percentages of the MERIT-DEM classes, with the sum of vertical values equal to 100 (**d**); and of the 3DEP-1 classes, with the sum of the horizontal values equal to 100 (**c**). The sum of the values in the blue boxes is equal to 100, and so on for each row (**c**) and column (**d**).

To assess the sensitivity of pattern delineation of the geomorphological forms derived from MERIT-DEM and 3DEP, we compare the geomorphological classification agreement for an area of 300 × 300 km (3000 × 3000 picels at 100 m spatial resolution) in South Dakota, USA. Figure 5 shows two raster plots (*a,b*) with a similar pattern at large scale but when observed in finer detail, there are differences in the classification at the pixel level (see Fig. 5a,b magnified circle). A common way to analyse the differences between two classifications is the calculation of a so-called confusion (or error) matrix^62^. The confusion matrix displays the probabilities with which pixels belonging to a certain class in one product appear in the same or a different class in the compared product. A confusion matrix can therefore be used to illustrate not only the degree to which the two classifications agree but also reveal how likely a class is misclassified.

In order to allow a numerical comparison of the geomorphological classifications, we calculate two confusion matrices among the 10 classes in each product (see Fig. 5). One is expressed as percentage of MERIT-DEM classes such that the sum within each row is equal to 100 (see Fig. 5c). Considering 3DEP-1 as a reference product, this would give the “user accuracy” of the MERIT-DEM geomorphometric classes. For instance, almost 70% of the *flat* pixels in MERIT-DEM also appear to be *flat* in 3DEP-1, while 10% are overlapping either with foothill or shoulder which are indeed likely spatial neighbours to *flat*. This is possibly an indication for some co-registration or interpolation issues affecting the two products.

The other matrix is calculated to display the percentages with respect to the 3DEP-1 classification, i.e. each column will sum up to 100 (see Fig. 5d). It shows the likelihood with which a 3DEP-1 class appears in the same or other classes of MERIT, which is called “producer accuracy”, e.g. 86% of *flat* pixels in 3DEP-1 are also correctly classified as *flat* in MERIT. Additionally, the most likely confusion here is again with *foothill* or *shoulder* classes (both around 6%), which support the above assumption of a co-registration issue. Another interesting finding is the widespread confusion between the *summit* and *ridge* class.

The congruence of *summit* pixels between the two products is in fact less likely than their respective confusion with *ridges* in the other. In addition, there are at least three times as many *summit* pixels detected in 3DEP-1 compared to MERIT. Similar anomalies occur for the morphologically inverse classes *depression* and *valley*. The reason for these results could either be due to an increased richness of detail (or actual resolution) offered by 3DEP-1 or a higher level of noise (though the latter being less likely given its high level of detail). Nevertheless, this preliminary analysis shows that the underlying DEM data yield significantly different geomorphometric characteristics and that the confusion matrix allows these differences to be numerically expressed.

### Comparing MERIT-DEM vs LiDAR elevation

**Fig. 6:**
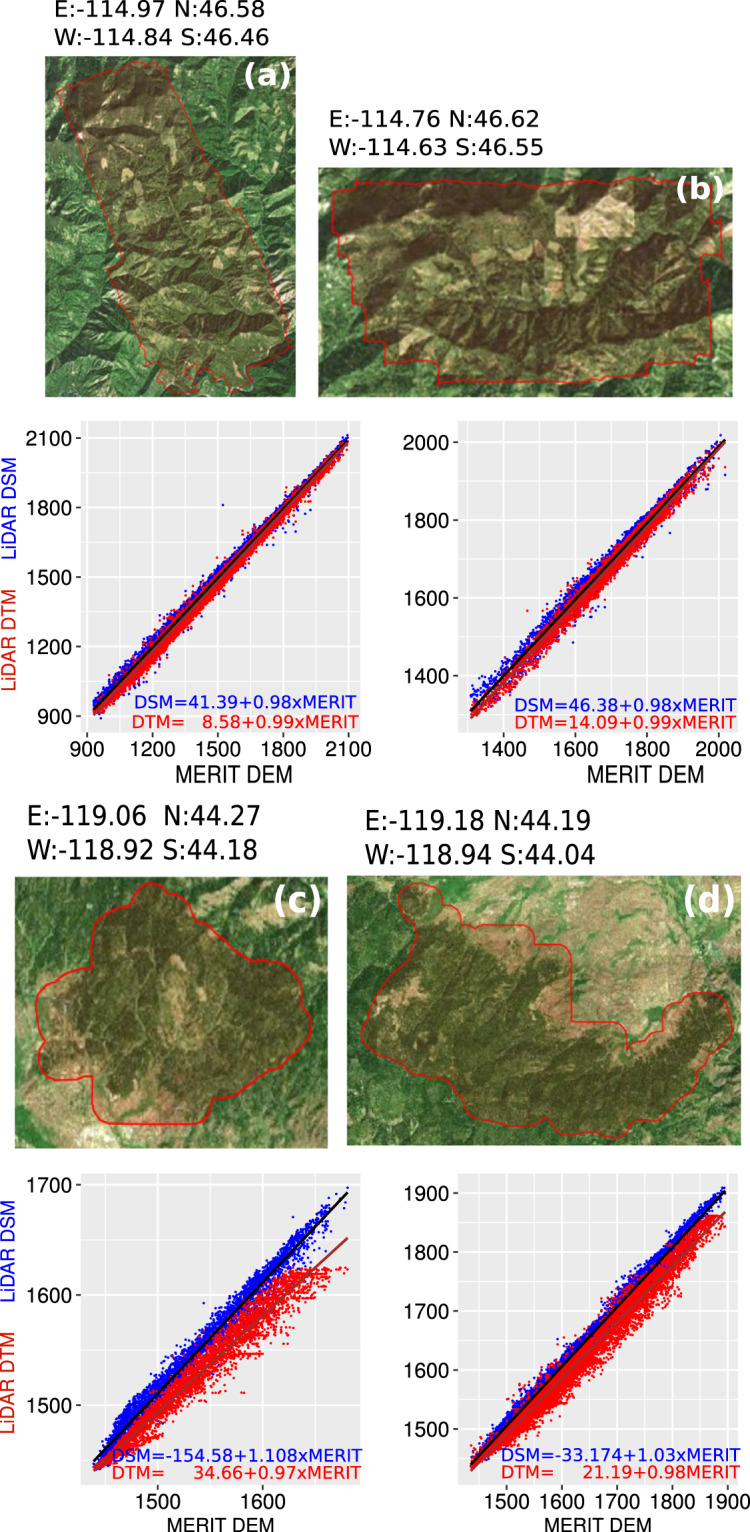
MERIT-DEM vs LiDAR-DEM. Comparison of MERIT-DEM with the LiDAR DSM and LiDAR DTM for four study areas, represented by their scatter-plots and their relative linear models.

Last return points in LiDAR data, which penetrate dense vegetation, are used to extract the DTM, whereas the first returns that hit the canopy of vegetation are used to derive the DSM. In our case, the LiDAR DTM and DSM were used to assess the quality of the tree height removal procedure carried out for the MERIT-DEM. Figure 6 reports the DTM vs. MERIT-DEM (red points) and the DSM vs. MERIT-DEM (blue points) of four study areas in USA with a high forest cover. In the four scatter plots, it is possible to distinguish the height difference between the DTM and DSM. The MERIT-DEM dataset has been corrected for the tree height bias^17^, and consequently the MERIT-DEM elevation values are expected to be closer to the LiDAR DTM than those of the LiDAR DSM. The LiDAR DTM vs. MERIT, and the LiDAR DSM vs. MERIT-DEM differences are analytically quantified by the linear model depicted in the scatter plot of Fig. 6. Where there is no vegetation or low vegetation (e.g. agricultural plains - Fig. 6c,d; bare ground mountain tops - Fig. 6a,b), the DTM and DSM have almost identical values, denoted by an overlap of red and blue points. The presence of similar elevation values in the DTM and DSM causes a convergence of the linear models (blue and red lines; the linear model functions are reported at the bottom of each scatter plot). The convergence in the lower parts of the plot (lower elevations) corresponds with the landscape (flat areas) and the absence of forest cover, which contributes to very similar values in the DTM and DSM. Whereas, at higher elevations, where there is increased forest cover, there is a greater difference between the DTM and DSM. This phenomena is more evident in Fig. 6c,d. On the contrary, Fig. 6a,b show a more parallel trend of blue and red lines, which is due to forest cover that is more equally distributed along the relief. It is important to note that the the scatter plots are plotted with different elevation ranges (x- and y-axis), and therefore the deviation of the red and blue line appears more evident in plots with lower elevation range (Fig. 6c). The regression coefficients for 3DEP-1 DTM vs. MERIT-DEM are slightly below 1 with an intercept value ranging from 8 to 34 m. On the other hand, the 3DEP-1 DSM vs. MERIT-DEM correlation has a regression coefficient larger than 1 and an intercept ranging from -154 to 46. The mean difference between DTM and DSM for Fig. 6a–d study areas ranges from 12 to 21 m. Overall, these values demonstrate the strong correlation between the MERIT-DEM and the LiDAR DTM.

### Identification of artefacts

It is important to note that the use and application of MERIT is based on the understanding that while it currently represents the best quality DEM available, it still contains errors and artefacts, which have not been corrected within the context of this research, as this was considered beyond the scope of the specific research objective. Consequently these errors cascade into the new Geomorpho90m dataset. The effect of these errors are mainly due to stripes that were recurrent in the AW3D product. These are visible in flat areas, where the artefact error is larger than the delta between pixels (e.g. slope). For instance, stripe artefacts can be found at the following locations in western Russia^63^ as well as in central Russia^64^.

## Usage Notes

The Geomorpho90m dataset^14–16^ is the first global scale geomorphometric layer product at a spatial resolution of 3 arc-seconds (~90 m) and 100 m, and has the potential to open new research avenues for a variety of research disciplines that require detailed geomorphometric and land surface information. For instance, the new layers can provide essential input data for analysing and modelling patterns and processes in physical geography, hydrological and climate science, land-use and land cover change, ecology, biogeography, conservation and biodiversity science. For hydrological applications in particular, the Geomorpho90m dataset creates, in combination with other environmental layers, the basis for computing freshwater-specific variables as per the procedure described in^65^. Most importantly, the product’s global coverage ensures that a standardised set of geomorphometric layers that enable comparative analyses across continents. Additionally, the newly-developed layers provide an update for the previous GMTED2010-derived topographic variables, as described in Amatulli *et al*.^5^. The new Geomorpho90m dataset can be considered more accurate in terms of its spatial resolution than the one by Amatulli *et al*.^5^ (90 vs. 250 m) and reduces potential residual errors from the corrections applied in the underlying MERIT-DEM dataset^17^. Therefore, the Geomorpho90m dataset provides deeper insights into the scale-dependency of geomorphometric characteristics across a wide variety of land surface-based studies.

The Geomorpho90m dataset^14–16^ provides an update of the “GMTED2010-derived topographic variables” described at Amatulli *et al*.^5^.

## Data Availability

**Geomorphometric layer computation** Prior to computing the geomorphometric layers, we reprojected the DEMs (MERIT, 3DEP-1, LiDAR DMS and DTM) to the Equi7 projection with a cell size 100 m (the projection parameters are available from^38^). To harmonise the different spatial grains, we used a bilinear algorithm implemented in *gdalwarp* within the open-source Geospatial Data Abstraction Library (GDAL). We kept the seven projection zones as defined in^36^, and employed the T6 tiling method to allow parallel and distributed processing of our work-flow. Tile size was 600 × 600 km where we buffered the borders by 401 km to avoid border artefacts between tiles. These overlapping and duplicate grid cells were removed when merging all tiles to seamless, continental maps. These large tile size increments were needed to avoid border effects, especially for multiscale deviation and multiscale roughness. We used the following open source software packages to compute the geomorphometric layers (Table 1 reports the software and the specific commands used for each derived variable calculation): Geospatial Data Abstraction Library (GDAL),version number 2.1.2^66^. Geographic Resources Analysis Support System software (GRASS), version number 7.3.0^67^. Whitebox Geospatial Analysis Tools (Whitebox GAT), version number 3.3.0^68^. Processing Kernel for geospatial data (Pktools), version number 2.6.3^69,70^. All of these tools provide fast and scalable computation features and functions for raster-based workflows that are easily automated using a scripting language, such as Bash or Python^71^. They also allow for the processing of very large datasets owing to efficient algorithms and optimised memory management. After computing all geomorphometric layers within the Equi7 projection, the layers were reprojected back to the WGS84 coordinate reference system (EPSG:4326 code^35^) with a bilinear algorithm (or near for categorical variables) implemented in GDAL. This reversion of Geomorpho90m to WGS84 allows for the data to be seamlessly integrated with a broad set of global datasets. We used a tiling system identical to MERIT-DEM, in terms of dimension and nomenclature, i.e. 5 × 5 degree tiles with 6000 × 6000 cells each, to ensure data integration and comparisons with the original MERIT-DEM. All calculations were processed in parallel using open-source software at the Center for Research Computing, Yale University. **LiDAR data processing** Two common products that can be extracted from LiDAR data are: DTM and DSM. The DTM is generated using ground echoes from the LiDAR point cloud, in conjunction with an interpolation technique^72^. The approach employed in this paper for calculating the DTMs used the LiDAR processing tools found in the pktools software^70^. This is a two-stage approach, where the first stage uses a minimum composite rule, which retains the LiDAR pulse for the minimum height of each cell. pklas2img -a_srs EPSG:26911 -dx 100 -dy 100 -comp min -i input.las -o dtm_min.tif For the second stage, the output is then filtered using a progressive morphological filter^73^, which uses an iterative filter based on increasing kernel sizes to remove non-ground points from the final DTM. pkfilterdem -f promorph -dim 3 -dim 11 -i dtm_min.tif -o dtm_min_promorph.tif The calculation of the DSM is more straightforward and uses either a maximum composite rule (pulse retention with the maximum height of the cell) or a defined percentile composite rule. In our case, we use the rule to retain the pulse with the value corresponding to the 95th percentile of all pulses within the cell. pklas2img -a_srs EPSG:26911 -dx 100 -dy 100 -comp percentile -percentile 95 -i input.las -o dsm2.tif The LiDAR projection parameters (EPSG code) were set to EPSG:26911 in accordance with the associated metadata. The DSM and DTM cell size was set to 100 m to allow a simple reprojection to Equi7, which enables a comparison with the other discussed layers.
